# Turning the respiratory flexibility of *Mycobacterium tuberculosis* against itself

**DOI:** 10.1038/ncomms12393

**Published:** 2016-08-10

**Authors:** Dirk A. Lamprecht, Peter M. Finin, Md. Aejazur Rahman, Bridgette M. Cumming, Shannon L. Russell, Surendranadha R. Jonnala, John H. Adamson, Adrie J. C. Steyn

**Affiliations:** 1 KwaZulu Natal Research Institute for Tuberculosis and HIV (K-RITH), K-RITH Tower Building Level 3, 719 Umbilo Road, Durban 4001, South Africa; 2 Department of Internal Medicine, University of Pittsburgh, 1218 Scaife Hall 3550 Terrace Street, Pittsburgh, Pennsylvania 15261, USA; 3 Tuberculosis Research Section, NIAID, NIH, 6610 Rockledge Drive, Bethesda, Maryland 20817, USA; 4 Department of Microbiology, University of Alabama at Birmingham, 1720 2nd Avenue South, Birmingham, Alabama 35294-2170, USA; 5 Centres for AIDS Research and Free Radical Biology, University of Alabama at Birmingham, 1720 2nd Avenue South, Birmingham, Alabama 35294-2170, USA

## Abstract

The *Mycobacterium tuberculosis* (Mtb) electron transport chain (ETC) has received significant attention as a drug target, however its vulnerability may be affected by its flexibility in response to disruption. Here we determine the effect of the ETC inhibitors bedaquiline, Q203 and clofazimine on the Mtb ETC, and the value of the ETC as a drug target, by measuring Mtb's respiration using extracellular flux technology. We find that Mtb's ETC rapidly reroutes around inhibition by these drugs and increases total respiration to maintain ATP levels. Rerouting is possible because Mtb rapidly switches between terminal oxidases, and, unlike eukaryotes, is not susceptible to back pressure. Increased ETC activity potentiates clofazimine's production of reactive oxygen species, causing rapid killing *in vitro* and in a macrophage model. Our results indicate that combination therapy targeting the ETC can be exploited to enhance killing of Mtb.

## Abstract (web summary)

Several inhibitors of the electron transport chain (ETC) are active against *Mycobacterium tuberculosis*. Here, Lamprecht *et al*. show that a combination of known inhibitors (bedaquiline, Q203 and clofazimine) displays synergistic effects on the *M. tuberculosis* ETC and improved antibacterial activity *in vitro*.

## Graphical abstract

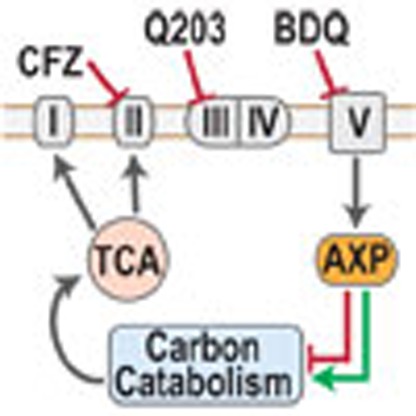

M*ycobacterium tuberculosis* (Mtb), the causative agent of tuberculosis (TB), kills more people than any other bacterium. TB control is threatened by the continued spread of drug resistance; multi-drug and extensively drug resistant Mtb require longer, more costly, treatment with multiple drugs causing worse side effects and have a lower likelihood of treatment success. The urgent need for better treatment options for drug resistant Mtb has led the World Health Organization to prioritize development of not only new individual antitubercular agents, but also new drug regimens^1^,^2^,^3^,^4^,^5^.

Mtb is an obligate aerobe, requiring the use of its flexible, branched electron transport chain (ETC) for energy production via oxidative phosphorylation (OXPHOS)^6^. Even during hypoxic non-replicating persistence, Mtb uses its ETC to dispose of reducing equivalents and maintain membrane potential^7^,^8^, reinforcing the importance of the ETC *in vivo*. The ETC-targeting diarylquinolone bedaquiline (BDQ) is the first novel antitubercular agent to be FDA approved in 40 years, and has sparked interest in ETC-targeting agents^9^, although some researchers express concern about the value of the Mtb ETC as a target^10^. BDQ inhibits ATP synthase (ETC Complex V) by binding to subunit c^11^, starving the bacteria of ATP^12^,^13^. BDQ has been thought to cause ‘back pressure' on the ETC, inhibiting proton pumping ETC complexes due to increased proton motive force (PMF), and slowing overall metabolic flux^6^,^14^,^15^. However, recently it has been proposed to act by allowing Complex V to act as a proton channel^16^. The imidazopyridine Q203, another new antitubercular drug, is currently in development. Q203 inhibits cytochrome bc_1_ (ETC Complex III) by binding to its QcrB subunit^17^, forcing the bacillus to use the less energetically efficient terminal oxidase, cytochrome bd. Both drugs cause several days of bacteriostasis and ATP depletion before becoming bactericidal. Clofazimine (CFZ) is an older antimycobacterial agent^18^ with good *in vitro* activity. CFZ shuttles electrons from the ETC enzyme type 2 NADH dehydrogenase (NDH2) to O_2_, generating bactericidal reactive oxygen species (ROS)^19^. Interest in CFZ for TB treatment continues as recent trials have evaluated CFZ in combination with other anti-tuberculosis drugs in animal^20^,^21^ models and in human^22^,^23^ clinical trials.

Energy production pathways are tightly regulated using multiple feedback loops to maintain energy homoeostasis^24^,^25^. Mtb undergoes metabolic remodelling in response to BDQ, although this has not been well-characterized^14^. Even less is known about Mtb's metabolic response to Q203 and CFZ. The combination of multiple feedback loops and a flexible ETC may cause complex and even surprising responses to perturbation of one part of the system.

To clarify the ETC's value as a drug target, Mtb's bioenergetics response to ETC targeting must be better understood. For this purpose, we use extracellular flux analysis technology^26^, inverted membrane vesicle (IMV) experiments, flow cytometry and time kill curves, with wild–type (wt) and selected mutant strains of Mtb, to investigate the direct effects of ETC-targeting drugs and the downstream repercussions of ETC perturbation. We also examine the effect of CFZ, Q203 and BDQ combinations on cellular toxicity, and Mtb killing in a macrophage infection model. Together, our data shed light into the complex effects of ETC targeting and identify potential strategies for combination-targeting of the ETC to achieve synergistic rapid killing.

## Results

### BDQ and Q203 increase Mtb respiration

**Figure 1:**
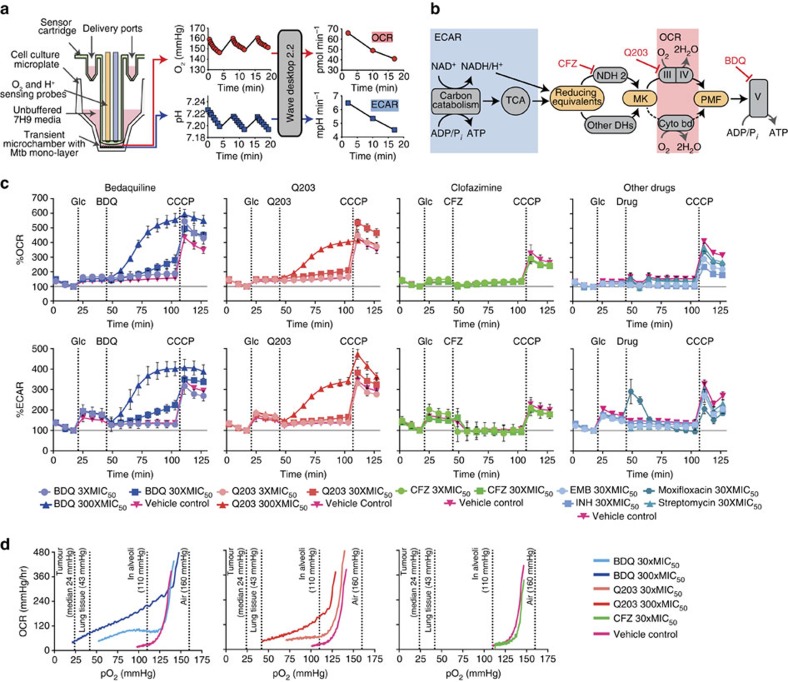
Diagram of the Seahorse XF Analyzer, its function and the initial bioenergetics analysis of Mtb in the presence of the ETC inhibitors. (**a**) Compounds are delivered into microplate wells via drug ports. When the probe is lowered, a transient microchamber is formed above a monolayer of bacilli. Dissolved O_2_ and pH are monitored by sensing probes. Oxygen consumption rate (OCR) and extracellular acidification rate (ECAR) are calculated from these measurements by the instrument software. (**b**) ECAR represents carbon catabolism and TCA cycle activity, which produce reducing equivalents that enter the ETC. Reducing equivalents pass through NDH2 or other dehydrogenases (DHs) to the menaquinone pool (MK), and then through Complexes III (cytochrome bc_1_) and IV (cytochrome aa_3_), or through cytochrome bd to O_2_. This contributes to the PMF, which powers ATP synthesis by Complex V (ATP synthase). CFZ acts on NDH2. Q203 inhibits Complex III. BDQ inhibits Complex V. (**c**) Bioenergetic analysis of Mtb. At the indicated times, 2 g l^−1^ of glucose (Glc) was added, followed by BDQ, Q203, CFZ, or other drugs, followed by the uncoupler CCCP to stimulate maximum respiration. BDQ and Q203, unlike CFZ or standard antimycobacterial drugs, induce an increase in bacterial respiration, above that of their respective vehicle controls. OCR and ECAR are indicated as a percentage of baseline values. Standard deviation of three replicate wells are indicated as calculated by the Seahorse XF Wave software. One representative experiment is shown; for ETC targeting drugs, at least three replicate experiments were performed. The following inter-experiment % CVs were calculated using Microsoft Excel (Microsoft Office 2010): basal OCR 47.2±5.2; % CV 11.1 (*n=6*), increased OCR after BDQ addition; 129.7±5.2, % CV 4.1 (*n=4*) and increased OCR after Q203 addition; 104.1±11.1, % CV 10.7 (*n*=4). The absolute value OCR profiles are shown in Supplementary Fig. 1. During optimization it was determined that fewer than 5% of the bacteria seeded into microplate wells were dislodged from the bottom during the experiment. (**d**) O_2_ depletion from microchamber sustained for 2 h. Each condition was repeated at least four times; representative traces are shown.

To determine the effect of BDQ, Q203 and CFZ on Mtb's bioenergetics, we used extracellular flux (XF) analysis technology (Fig. 1a) to measure Mtb's oxygen consumption rate (OCR) and extracellular acidification rate (ECAR) in real time as markers of OPHOS and carbon catabolism (Fig. 1b), respectively^27^. By adding inhibitors and substrates during each experiment, we can measure actual and maximum rates of activity of different components of energy-generating pathways.

Since Mtb is an obligate aerobe dependent on its ETC, we expected its bioenergetic profile to be affected by these ETC-targeting drugs. We treated Mtb H37Rv bacilli with BDQ, Q203, and CFZ while measuring OCR and ECAR in the XF96 Extracellular Flux Analyser^27^,^28^. The OCR measurements are reported as percentage of basal levels in Fig. 1c; absolute value OCR profiles for BDQ, Q203 and CFZ are contained in Supplementary Fig. 1. To measure basal and maximum rates of activity of different components of energy-generating pathways, we first added either glucose (Fig. 1c), palmitate, or lactate (Supplementary Fig. 2); then an ETC-targeting drug; and finally the uncoupler carbonyl cyanide *m*-chlorophenyl hydrazone (CCCP). CCCP depolarizes the cell membrane; this dose was optimized to increase respiration and carbon catabolism to the maximum sustainable by the bacterium. Due to limitations of solubility and the instrument's drug injection mechanism, CFZ could only be used up to a maximum concentration of 30 × its MIC_50_ in the well. CFZ, at both 3 and 30 × the MIC_50_, had little effect on bioenergetics compared with its vehicle (dimethylsulphoxide (DMSO)) control. Given that BDQ and Q203 are inhibitors of ETC Complexes V and III respectively, we expected them to inhibit respiration. To our surprise, OCR for both BDQ- and Q203-treated cells increased in a dose-dependent fashion to well above the vehicle control (assay media). ECAR also increased for both BDQ- and Q203-treated cells, indicating an increase in carbon catabolism in response to inhibition of OXPHOS.

To determine if this might be a general response of Mtb to antibiotic treatment, we repeated the above experiments with several first- and second-line antimycobacterial drugs. None of these drugs caused an increase in OCR (Fig. 1c). Thus, the BDQ- and Q203-mediated increase in respiration is not a general response to antibiotic treatment.

To establish whether this increase in OCR was a transient response to the onset of drug treatment, we measured OCR and ECAR for 16 h after drug addition (Supplementary Fig. 3). Both BDQ- and Q203-treated cells maintained an increased OCR and ECAR above vehicle-treated control for the duration of the experiment. Thus, BDQ and Q203 cause a lasting increase in respiration.

In summary, these experiments show that BDQ and Q203, unlike CFZ or standard antimycobacterial drugs, dramatically increase bacterial respiration, above that of their respective vehicle controls, in the presence of several different carbon sources. We subsequently performed a series of experiments to determine the mechanisms by which these two drugs increase respiration and the possible significance of this phenomenon.

### Respiration is increased over a wide O_2_ range

We next asked whether our previous results might be an artifact of experiments performed in well-aerated media, which may not be representative of the lower O_2_ tensions (partial oxygen pressure (pO_2_)) that Mtb experiences during infection. To investigate the effects of these drugs under lower pO_2_ we performed an experiment in which the XF96 microchamber was sustained for a 2-hour period, allowing the bacilli to partially deplete O_2_ from their microchambers (Fig. 1d). Although quantitative analysis of this data is limited by uncertainty regarding diffusion rates of oxygen into the microchambers, qualitative comparisons of treated and untreated wells revealed that, CFZ (at 30 × the MIC_50_) had minimal effect on OCR. In stark contrast, BDQ- and Q203-treated cells continued to consume O_2_ at a higher rate, depleting considerably more O_2_. Thus, BDQ and Q203 increase respiration over a range of physiologically relevant O_2_ concentrations^29^.

### BDQ and Q203 do not increase membrane proton conductance

**Figure 2:**
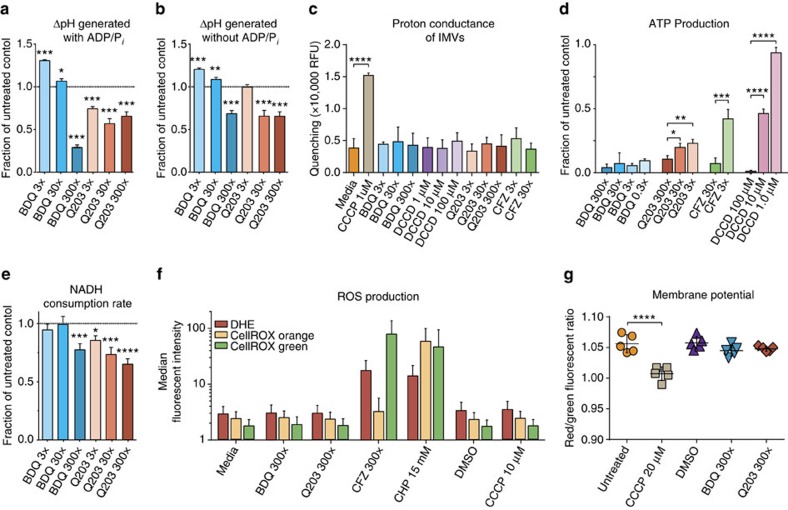
IMV, ROS and membrane potential experiments. ΔpH measured by quenching of fluorescent probe ACMA, ATP production measured with luciferase/luciferin system, the rate NADH consumption was monitored at 340 nm, ROS production via flow cytometry with ROS-sensitive dyes dihydroethidium (DHE), CellROX Orange and CellROX Green, and bacterial membrane potential with the BacLight^TM^ Membrane Potential kit via flow cytometry. (**a**) ΔpH generated by IMVs in the presence of NADH, with ADP and phosphate in the reaction buffer. (**b**) ΔpH generated by IMVs in the presence of NADH, without ADP or phosphate. (**c**) Proton conductance of IMVs. ΔΨ was generated by valinomycin treatment of IMVs in potassium-free buffer, ΔpH generation was taken as an indication of membrane permeability to protons. (**d**) ATP production by IMVs provided with NADH. (**e**) Rate of NADH consumption by IMVs in the presence of saturating concentrations of NADH (250 μM). (**f**) Median fluorescent intensity of ROS-sensitive dyes in treated Mtb mc^2^6230 cells. (**h**) Shifts in the red/green median fluorescent intensity ratio after control and drug addition shows that neither BDQ nor Q203 have a measurable effect on Mtb membrane potential, whereas CCCP does. Error bars are standard deviations of three replicate experiments, except for subpanel (**f**), for which the interquartile range of a single experiment is shown, and (**g**), for which five biological replicates were used. *P*-values were determined by one-way analysis of variance using GraphPad Prism 6.05. *P<0.05, **P<0.005, ***P<0.0005 and ****P<0.0001.

To determine if BDQ and Q203 might increase OCR by acting as a classical uncoupler, allowing H^+^ to more move freely across the membrane, we isolated Mtb mc^2^6230 IMVs. We used the fluorescent dye 9-amino-6-chloro-2-methoxyacridine (ACMA) to measure the ΔpH generated by the ETC using NADH as an electron donor (Fig. 2a,b). High doses of BDQ and Q203 reduced the ΔpH generated by IMVs using NADH, consistent with either decreased pumping of protons out of the IMV or increased conductance allowing more flow of protons into the IMV. To distinguish these cases, we measured the H^+^ conductance of IMVs incubated with BDQ, Q203, and CFZ. None of these compounds increased proton conductance under these conditions, although the protonophore CCCP did (Fig. 2c). Thus, the increase in respiration caused by BDQ and Q203 cannot be explained by directly increasing proton conductance. To confirm that our IMV model captures the effect of these drugs on the ETC, we measured also ATP production using a luciferase system. ATP production was inhibited by BDQ, Q203 and CFZ (Fig. 2d).

### Q203 and BDQ moderately reduce maximum ETC flux in IMVs

Although one might expect inhibition of ETC complexes to decrease electron flux, the increase in OCR in whole bacilli suggests increased ETC flux. To clarify this, we measured the maximum rate of NADH consumption of drug-treated IMVs. Q203 and the higher concentrations of BDQ significantly decreased the rate of NADH consumption (Fig. 2e) with a modest inhibition of ∼25%. Thus, while neither BDQ nor Q203 directly increase the reaction kinetics of ETC complexes, neither did they substantially decrease maximum flux of the ETC between NADH and O_2_. Given the increase in respiration seen in whole cells, this data suggests that in whole cells not artificially provided with a supraphysiological excess of NADH, the ETC does not reach its maximum flux and is not the rate-limiting step in overall metabolism under our conditions.

### BDQ and Q203 do not cause an increase in ROS production

We next asked whether the BDQ and Q203-triggered increase in OCR is due to the conversion of oxygen to ROS. We reasoned that disruption of the ETC might result in non-enzymatic transfer of electrons to O_2_, producing superoxide (O_2_^·−^). This would allow the same number of electrons to consume more O_2_ than when O_2_ is fully reduced to H_2_O. We thus measured ROS production of treated Mtb mc^2^6230 using three ROS-sensitive fluorescent dyes by flow cytometry (Fig. 2f). CFZ was used as a positive control given its known ROS generating activity. No increase in ROS was observed with BDQ or Q203. BDQ and Q203's increase in respiration therefore cannot be explained by conversion of large quantities of O_2_ directly to ROS. These data suggest that O_2_ is fully reduced to H_2_O, and the increase in respiration corresponds to an equal increase in electron flux through the ETC.

### BDQ and Q203 do not alter Mtb membrane potential

We further investigated whether the increase in OCR after BDQ and Q203 addition to whole cells might be due to an increase in proton conductance causing membrane depolarization, as seen with classical uncouplers. We used flow cytometry with the fluorescent dye 3,3′-diethyloxacarbocyanine iodide (DiOC_2_(3)) to measure the membrane potential of Mtb cells treated with Q203, BDQ; CFZ could not be assessed due to its fluorescence (Supplementary Fig. 4e). Gating strategies, mean fluorescent intensities and shifts in these mean fluorescent intensities after drug treatment are shown in Supplementary Fig. 4. Neither Q203 (300 × MIC_50_), BDQ (300 × MIC_50_), nor DMSO caused a change in membrane potential compared with the untreated control (Fig. 2g); although, as expected, the classical uncoupler CCCP did reduce the membrane potential. Given the limit of detection for membrane changes using this technique, we cannot rule out a smaller change in membrane potential than is caused by CCCP. This is consistent with our IMV experiments and previously published data^14^,^16^. Thus, the increase in OCR caused by these drugs cannot be explained by a severe membrane depolarization as is observed with classical uncouplers.

### Q203 induces rapid rerouting of the Mtb ETC electron flux

We next investigated whether the OCR increase is predominantly mediated by cytochrome bd. Q203 inhibits the cytochrome bc_1_/aa_3_ complex. BDQ has been assumed to exert back pressure on the ETC proton pumping complexes^6^,^14^,^15^, which suggests that BDQ inhibits the cytochrome bc_1_/aa_3_ complex more than bd, due to cytochrome bd's lower contribution to PMF. We, therefore, hypothesized that treatment with either BDQ or Q203 would reroute electron flux away from cytochrome bc_1_ and toward the other terminal oxidase, cytochrome bd.

**Figure 3:**
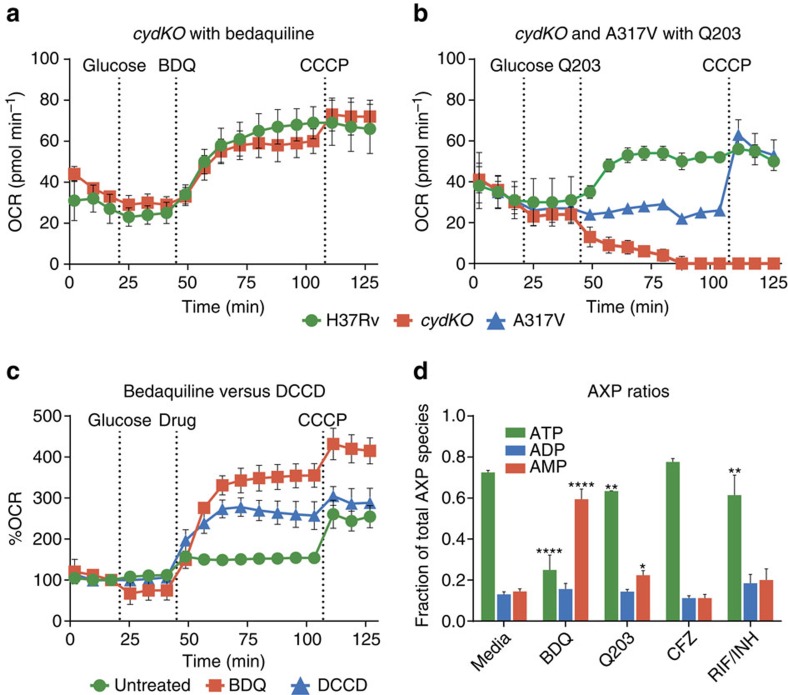
Bioenergetic analysis of Mtb H37Rv, cytochrome bd knockout (*cydKO*), and *cydKO* strain with Q203 resistance SNP (A317V) to ETC drugs. At indicated times, 2 g l^−1^ glucose was added, followed by either Q203, BDQ, or DCCD, followed by 2 μM CCCP. (**a**) 300 × MIC_50_ BDQ treatment has a very similar effect on H37Rv and *cydKO*. (**b**) 300 × MIC_50_ Q203 treatment causes H37Rv OCR to increase, however *cydKO* OCR drops to zero, while A317V OCR is unchanged. The untreated media OCR profiles of the Mtb H37Rv, *cydKO* and A317V strains are provided in Supplementary Fig. 4. (**c**) 100 μM of the ATP synthase inhibitor DCCD causes a similar OCR increase to 30 × MIC_50_ BDQ, although somewhat less in magnitude at these concentrations. (**d**) H37Rv Mtb was cultured for 24 h with 30 × MIC_50_ of BDQ, Q203, CFZ, a RIF/INH combination, or media control. Relative abundance of ATP, ADP, and AMP was measured by LC-MS/MS. Standard deviations of three technical replicates are shown, all experiments were performed at least twice. *P<0.05, **P<0.005 and ****P<0.0001 (one-way analysis of variance using GraphPad Prism 6.05).

We tested this hypothesis using wt Mtb, an Mtb cytochrome bd knockout (*cydKO*), and a *cydKO* mutant with a Q203-resistant single-nucleotide polymorphism (SNP). This A317V SNP is in the Q203-binding pocket on the QcrB subunit, and confers high level Q203 resistance^30^. BDQ had a similar effect on both wt and *cydKO* strains (Fig. 3a). Q203 increased the OCR of wt Mtb as before, however it rapidly decreased the OCR of Mtb *cydKO* to zero (Fig. 3b). Q203 did not affect *cydKO* A317V. The OCR profiles of the untreated strains are shown in Supplementary Fig. 5.

Thus, Q203 is able to fully inhibit electron flux through cytochrome bc_1_, as shown by the complete cessation of respiration in Q203-treated *cydKO*. Electron flux quickly reroutes to cytochrome bd if it is present, which consumes O_2_ at a higher rate than at baseline. In the QcrB SNP resistant strain, Q203 has no effect, demonstrating that the previously described changes are not due to off-target effects. BDQ increases OCR similarly in both wt and Mtb *cydKO.* Thus, BDQ's increase in respiration can be fully mediated by cytochrome bc_1_/aa_3_.

### DCCD inhibition of ATP synthase increases respiration

We next asked whether the BBQ induced increase in OCR is particular to its interaction with ATP synthase. We tested the bioenergetics of Mtb bacilli treated with the ATP synthase inhibitor *N,N*-dicyclohexylcarbodiimide (DCCD) and found that DCCD caused an increase in OCR (Fig. 3c). This suggests that an increased OCR may be a general response of Mtb to inhibition of ATP synthase.

### BDQ and Q203 deplete ATP

We hypothesized that the BDQ and Q203-induced increase in respiration might be a response to depletion of ATP, representing an attempt to increase the activity of energy-generating pathways to restore energy homoeostasis. This would predict that the severity of ATP depletion correlates with OCR increase. To test this hypothesis, Mtb bacilli were cultured in the presence of these drugs, and adenosine phosphate species (AXP) were measured using liquid chromatography tandem mass spectrometry (LC-MS/MS). We found that ATP was depleted most severely by BDQ, to a lesser extent by Q203, and not at all by CFZ (Fig. 3d), mirroring the degree of respiratory stimulation. This is consistent with respiration increases being driven by ATP depletion.

### BDQ increases respiration independent of cytochrome bc_1_

**Figure 4:**
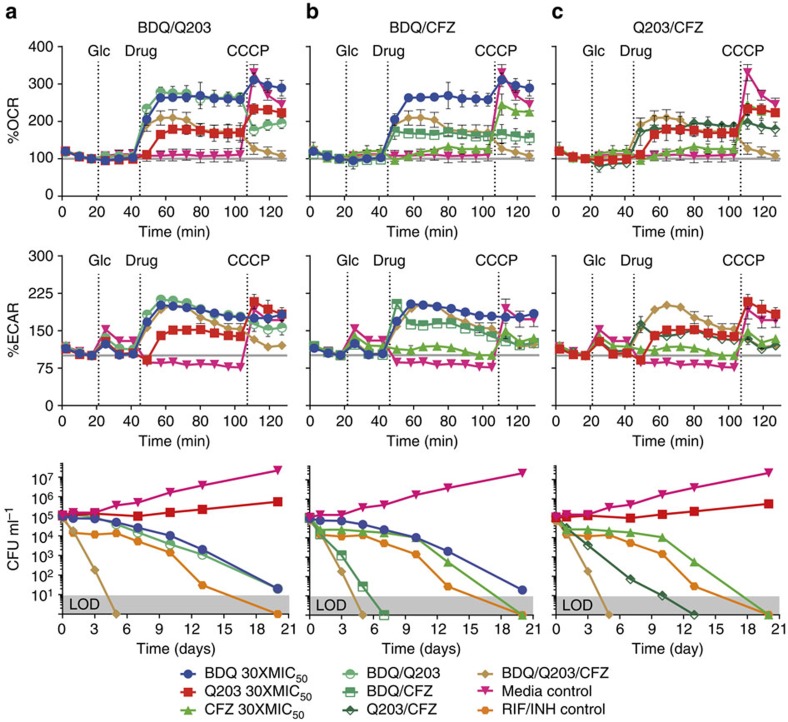
Analysis of combination treatment of Mtb H37Rv. Indicated additions are 2 g l^−1^ glucose (Glc), drug combination, and 2 μM CCCP as an uncoupler to stimulate maximum respiration. OCR and ECAR are shown as a per cent of baseline values with standard deviation of three replicate experiments performed. Kill curves were constructed from CFUs measured over 20 days of treated cells grown in 7H9 with 2 g l^−1^ of glucose and 0.01% tyloxopol. Limit of detection (LOD) is 10 CFU per ml. All drugs were added at 30 × MIC_50_ values. Two separate kill curve experiments were performed with the same results, representative data is shown. (**a**) BDQ, Q203, BDQ/Q203, and BDQ/Q203/CFZ combinations. (**b**) BDQ, CFZ, BDQ/CFZ and BDQ/Q203/CFZ combinations. (**c**) Q203, CFZ, Q203/CFZ, and BDQ/Q203/CFZ combinations.

We next asked whether BDQ's increase in respiration depends on which terminal oxidase is used by the cell. We had previously seen that BDQ causes a similar increase in OCR in wt and in Mtb *cydKO* (Fig. 3a), indicating that the full electron flux can flow through cytochromes bc_1_ and aa_3_. To assess the effect of BDQ treatment when electron flux is forced to flow through cytochrome bd, we measured the bioenergetics of BDQ/Q203 co-treated cells on the XF96. BDQ/Q203 co-treatment causes an increase in OCR and ECAR similar to that of BDQ treatment alone, although uncoupled respiration is reduced (Fig. 4a). Thus, BDQ's increase in respiration can be fully mediated by cytochrome bd.

### Combination-targeting of the Mtb ETC

We next investigated whether the similar bioenergetic profiles of BDQ and BDQ/Q203-treated cells corresponded to similar kill kinetics. We thus constructed kill curves by counting colony-forming units (CFUs) of co-treated cells over 20 days (Fig. 4a, bottom graph). The BDQ/Q203 combination kills Mtb at the same rate as BDQ alone.

To ascertain the effects of combinations of CFZ with BDQ and Q203, we measured the bioenergetics of combination-treated cells with the XF and also constructed CFU kill curves over 20 days (Fig. 4b,c). CFZ in combination with BDQ and/or Q203 increases OCR and ECAR slightly less than BDQ and/or Q203 alone. CFZ kills Mtb synergistically with either BDQ or Q203. The CFZ/Q203/BDQ triple combination kills even faster, with no detectable viable bacilli after 5 days. All CFZ-containing combinations kill faster than the combination of RIF and INH at equivalent MIC multiples, and cause immediate, rapid, steady killing, without an initial bacteriostatic phase. The rate and immediate onset of killing with CFZ suggests a different mechanism from the ATP depletion attributed to BDQ or Q203 alone.

### Rapid killing correlates with reductive stress

We hypothesized that increased rapid killing of CFZ-containing combinations is caused by increased ROS production by CFZ. We reasoned that increased ETC flux caused by BDQ or Q203 suggests a more reduced ETC, possibly potentiating CFZ's diversion of electrons to ROS production, which would explain the increased rapid bactericidal activity.

**Figure 5:**
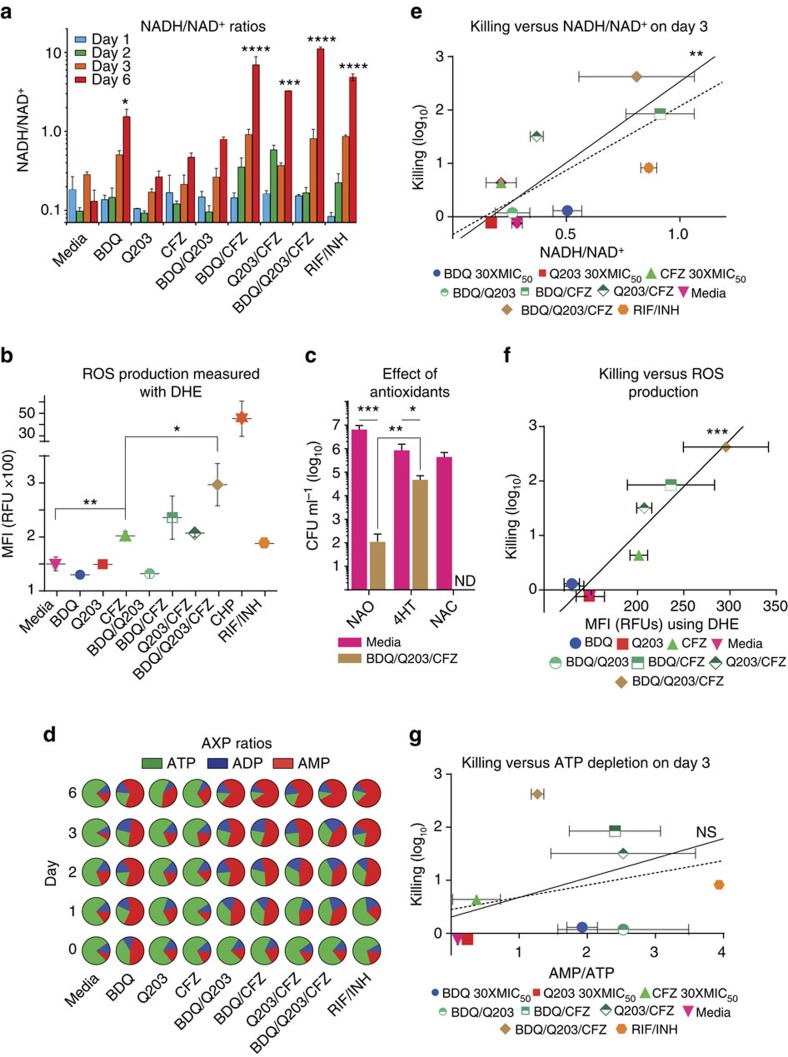
Assessment of contributors to rapid synergistic killing of CFZ-containing combinations. (**a**) NADH/NAD^+^ ratios of cells cultured with indicated drug combinations at 30 × MIC_50_, as measured by LC-MS/MS. (**b**) ROS produced by cells cultured with indicated drug combinations at 30 × MIC_50_, as measured by flow cytometry with DHE. (**c**) The killing efficacy of the BDQ/Q203/CFZ under three different conditions after 5 days of treatment: no antioxidant (NAO), 4-Hydroxy-Tempo (4HT) and *N*-acetyl-cysteine (NAC). Representative data is shown of three independent experiments performed. Limit of detection (LOD) is 10 CFU per ml. ND, not detected. (**d**) ATP, ADP, and AMP ratios of cells cultured with indicated drug combinations at 30 × MIC_50_, as measured by LC-MS/MS. All experiments were performed in triplicate; error bars indicate standard deviation of measurements. *P<0.05, **P<0.005, ***P<0.0005 and ****P<0.0001 (one-way analysis of variance using GraphPad Prism 6.05). Correlation between reduction in CFU per ml (log_10_) at Day 3 as a measure of early, rapid killing, and (**e**) reductive stress, (**f**) oxidative stress, and (**g**) ATP depletion stress. Correlations are shown for all drugs (dotted line), as well as for ETC-targeting drugs only (solid line). Excluding RIF/INH control, which is expected to have a different mechanism of action, linear correlation statistics were determined using GraphPad Prism 6.05 and are as follows: (**e**) *R*^2^=0.83, *P*=0.002; (**f**) *R*^2^=0.91, *P*=0.0002; (**g**) *R*^2^=0.137, *P*=0.4.

To test this hypothesis, we first treated wt Mtb bacilli with all drug combinations, measuring NADH and NAD^+^ levels over 6 days via LC-MS/MS (Fig. 5a). By day 3 it was clear that more reducing equivalents had been accumulating in more lethal combinations (Fig. 5e). This is consistent with the hypothesis that BDQ and Q203 drive the cell into a more reduced state that potentiates CFZ's ROS production.

### Rapid killing correlates with ROS production

To evaluate whether CFZ-containing combinations increase ROS production, we measured ROS in Mtb using the fluorescent dye dihydroethidium (DHE) via flow cytometry (Fig. 5b). BDQ and Q203 treatment did not increase ROS as compared with vehicle control, however the BDQ/Q203/CFZ combination increased ROS significantly more than CFZ alone. Thus ROS production is highly correlated with rapid killing (Fig. 5f), suggesting that the rapid, synergistic killing of CFZ-containing combinations is mediated by increased ROS.

### Rapid killing is mitigated by 4-hydroxy-TEMPO

To test the hypothesis that the rapid killing was caused by ROS toxicity, we investigated whether the antioxidants 4-Hydroxy-2,2,6,6-tetramethylpiperidyl-1-oxy (4-hydroxy-TEMPO, 4HT) and *N*-acetyl-cysteine (NAC) are protective against the BDQ/Q203/CFZ triple combination. Mtb cultures were independently drug treated for 5 days in the presence and absence of 4HT or NAC (Fig. 5e). The antioxidants caused only modest inhibition of Mtb growth compared with the no antioxidant control. 4HT, but not NAC significantly protected against BDQ/Q203/CFZ combination by ∼3 log_10_ (*P*<0.005; Fig. 5c). Given that 4HT is selective towards superoxide (O_2_^·–^)^31^, and NAC is more effective against hydroxyl radicals^32^,^33^, this is consistent with the triple combination killing by O_2_^·–^.

### Rapid killing does not correlate with ATP depletion

Because BDQ and Q203 are thought to kill by ATP depletion, we next evaluated the competing hypothesis that CFZ-containing combinations rapidly kill via more severe ATP depletion. To test this hypothesis, we cultured Mtb with all drug combinations, measuring AXP levels over 6 days via LC-MS/MS. Adding CFZ to BDQ or Q203 did not increase ATP depletion (Fig. 5d), and rapid killing did not correlate with ATP depletion (Fig. 5g). This suggests that the rapid immediate killing of CFZ-containing combinations is not mediated by greater ATP depletion.

### Drug combinations are non-toxic in mammalian cell lines

**Figure 6:**
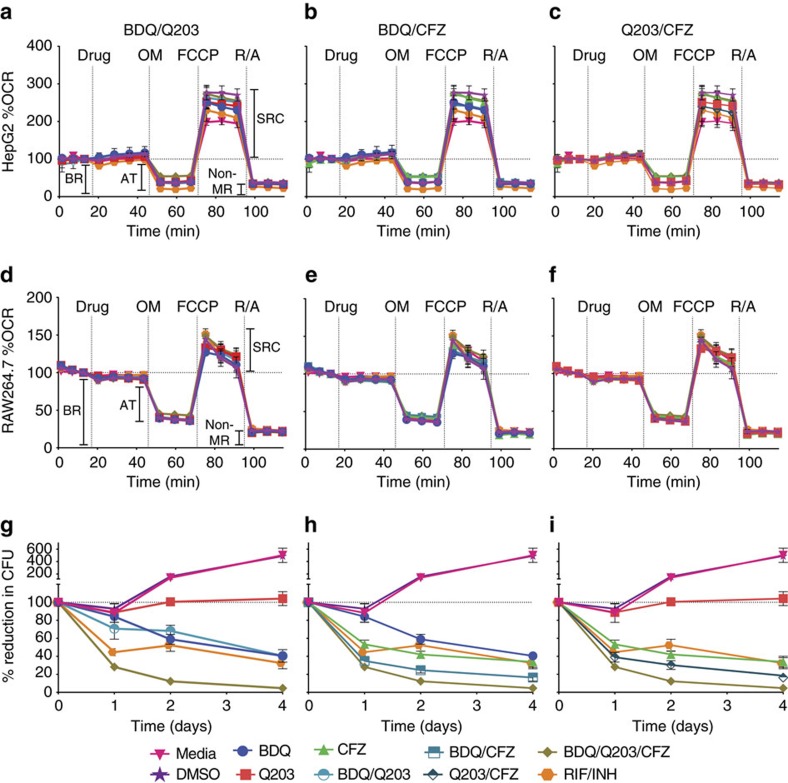
Analysis of the effects of different Mtb ETC targeting drug combinations on mammalian cell lines. Cell lines were treated with the ETC targeting drugs at 30 × MIC_50_ concentrations. Oligomycin was used at a concentration of 3.0 μM and 1.5 μM for HepG2 and RAW264.7 cells, respectively. FCCP was used at a concentration of 1.5 μM, and rotenone and antimycin A were both used at a concentration of 0.5 μM. HepG2 cells (**a**–**c**) and RAW264.7 cells (**d**–**f**) were seeded at a density of 25,000 and 65,000 cells per well, respectively. In both the HepG2 and RAW264.7 cell lines the Mtb ETC targeting drug combination had similar effects on the four bioenergetic parameters as observed in the controls. These parameters include basal respiration (BR), ATP turnover (AT, an indication of the amount of O_2_ consumed to produce ATP in the mammalian ETC), spare respiratory capacity (SRC, an indication of the cell's capacity to respond under stressful conditions) and non-mitochondrial respiration (non-MR, *e.g*. NADPH oxidase mediated reduction of O_2_ during the oxidative burst). Experiments were performed twice. Kill curves (**g**–**i**) were constructed by infecting 200,000 RAW264.7 cells per well with Mtb H37Rv at a MOI of 1 and drug treating at 30 × MIC_50_ concentrations, for 4 days. CFU data is presented as the mean percentage±s.d. reduction in CFU from Day 0 of three independent experiments performed in triplicate.

To determine the cytotoxicity of the different Mtb ETC targeting drug combinations towards HepG2 and RAW264.7 cell lines we preformed both bioenergetic (Fig. 6, panels a–e, respectively; as well as Supplementary Fig. 6) and MTT cell viability assays (Supplementary Fig. 7), and found no obvious cytotoxic effects. These results are discussed in full in the Supplementary Discussion.

### Enhanced Mtb killing in a macrophage infection model

Finally, knowing that these drug combinations do not affect mammalian cell bioenergetics or viability, we assessed how effectiveness in a macrophage infection model in three independent experiments. We infected RAW264.7 cells with Mtb H37Rv, treated with drug combinations, and measured CFUs for 4 days (Fig. 6, Panels g–i, representative CFU per ml shown in Supplementary Fig. 8). Consistent with the *in vitro* kill curves (Fig. 4), the BDQ/Q203/CFZ triple combination was the most effective in killing Mtb, causing a 95.4±2.7% reduction in CFUs over 4 days of treatment. The BDQ/CFZ and Q203/CFZ combinations resulted in an 83.6±4.4% and 81.5±1.5% reduction in CFUs over 4 days, respectively—with the RIF/INH combination only achieving a 67.7±2.7% reduction in CFUs in the same time. Thus, as was observed in *vitro*, adding CFZ to BDQ and/or Q203 leads to enhanced Mtb killing in a macrophage infection model.

## Discussion

**Figure 7:**
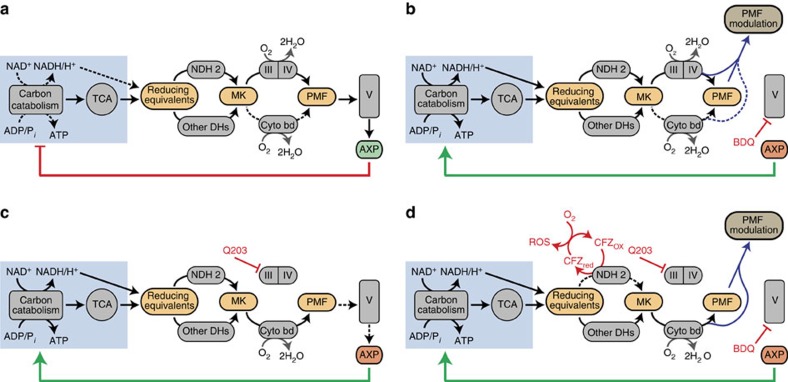
Proposed models of Mtb energy generation pathways under different drug treatment conditions. (**a**) In untreated Mtb the proton motive force (PMF) produced by the terminal oxidases is used by Complex V to produce ATP. AXP ratios allosterically regulate both glycolysis and the TCA cycle via feedback inhibition to maintain energy homeostasis. (**b**) In the presence of BDQ, Complex V and thus ATP production is inhibited. Although Complex V does not relieve the PMF, PMF is modulated by either intrinsic or extrinsic uncoupling, preventing back pressure. Without OXPHOS, ATP levels decline, relieving carbon catabolism and the TCA cycle of feedback inhibition. This increases production of reducing equivalents, which enter the ETC and increase OCR. As the feedback loop is broken by BDQ, this control system fails to regulate activity appropriately, and glycolysis and the TCA cycle accelerate to their maximum pace. (**c**) In the presence of Q203, Complex III is inhibited. Electrons reroute from the Complex III/IV pathway to the cytochrome bd pathway, which generates less PMF. This reduces the efficiency of OXPHOS, leading to declining ATP levels, a decrease in feedback inhibition of carbon catabolism and the TCA cycle, increased production of reducing equivalents and increased respiration. The feedback loop is still functional, and a new steady state is reached with reduced ATP levels. (**d**) In the presence of all three drugs, production of reducing equivalents is increased as discussed above. The increased flux of electrons through NDH2 enhances CFZ's ability to divert electrons to O_2_, causing the formation of toxic ROS.

Here we have shown that the Mtb ETC has a greater metabolic flexibility and ability to reroute around pharmacological inhibition than has previously been appreciated. Our data shows that, unlike in eukaryotes, back pressure does not substantially impede ETC activity; this suggests that an energy spilling pathway modulates PMF to prevent excessive accumulation. We propose a model whereby both BDQ and Q203 increase total ETC flux and respiration due to loss of feedback inhibition of carbon catabolism and the TCA cycle by ATP (Fig. 7b,c). Rather than suggesting that the ETC is an undesirable drug target, however, our results have demonstrated a manner to turn this plasticity against Mtb. The ability of Q203 to rapidly reroute ETC flux from cytochrome bc1/aa3 to cytochrome bd was a key discovery towards understanding this plasticity. By using respiratory inhibitors to rewire energy metabolism, we potentiate CFZ's production of bactericidal ROS. Notably, the efficacy of ETC targeting drug combinations *in vitro* was validated in a macrophage infection model, which showed no toxicity towards host cell lines. Overall, our findings reveal novel insights into the Mtb ETC as a drug target that can be effectively exploited for new therapeutic intervention strategies.

In our proposed model, the increase in respiration caused by BDQ, Q203 and DCCD occurs because decreasing ATP levels cause the cell to increase activity of energy-generating pathways in an effort to maintain homoeostasis (Fig. 7). Key enzymes in glycolysis and the TCA cycle are allosterically regulated by AXP species. This has been best characterized in *Escherichia coli*^25^; however, this regulation has been seen in other organisms^34^,^35^ including other *Corynebacterineae* such as *Corynebacterium glutamicum*^36^. This regulation by AXP plays the largest role in determining overall glycolytic flux^37^. As a result, ATP depletion in *E. coli* and *C. glutamicum* increases production of reducing equivalents, which enter the ETC, increasing oxygen consumption^38^,^39^. The action of this classic negative feedback loop normally serves to increase ATP production via both substrate level phosphorylation and OXPHOS, thus returning the cell to energy balance. Consistent with their mechanisms of action, BDQ, Q203 and DCCD decrease ATP production rates in IMVs (Fig. 2d), and deplete ATP levels in live cells (Fig. 3d). Thus, as long as the ETC can reroute around the inhibition of these compounds' targets, preventing them from imposing a substantial brake on glycolysis, the TCA cycle, or the ETC, there are strong theoretical grounds to expect all three to increase respiration, as we have observed. Interpreting the increase in respiration caused by these three compounds as a response to declining ATP levels provides a unifying explanation for the effect of all of them.

In our model, BDQ and Q203 drive the cell into very different states with respect to this feedback loop. Complete inhibition of Complex V, as can be achieved with BDQ and DCCD, breaks the feedback loop (Fig. 7b) whereby byproducts of carbon catabolism can be used to produce the ATP which suppresses further carbon catabolism. This causes a loss of feedback inhibition and subsequently severe dysregulation of metabolism results in the system accelerating to its maximum achievable rate. In contrast, complete inhibition of cytochrome bc_1_ with subsequent shift of electron flux to cytochrome bd, as can be achieved with Q203, decreases the efficiency of PMF generation and hence ATP production by a factor of three. Under our conditions, this results in a bacteriostatic phenotype with a moderately reduced intracellular concentration of ATP and approximately three times the ETC flux. Increasing ETC flux by a factor of three should compensate for the threefold fall in efficiency, ultimately resulting in similar levels of PMF generation and ATP production rates. This is all consistent with the key regulatory feedback loop continuing to work (Fig. 7c), albeit with lower efficiency, necessitating a lower ATP concentration to drive the increased metabolic rates required to maintain similar rates of ATP production.

Inhibition of Complex V has been proposed to inhibit the ETC by back pressure^6^,^14^,^15^, however, our data suggest that this does not occur in whole cells. Back pressure is the phenomenon widely seen in eukaryotic cells whereby a decrease in proton flow through ATP synthase leads to a buildup in PMF, causing the activity of PMF-generating ETC complexes to become less energetically favourable, thereby slowing or stopping ETC flux^40^. DCCD blocks Complex V's proton channel by covalent modification of carboxyl residues^41^. If Mtb was susceptible to back pressure, DCCD would slow or stop ETC flux and thus respiration. However, DCCD increases respiration (Fig. 3c), indicating a lack of susceptibility to back pressure. Intriguingly, an ATP synthase knockout mutant in *E. coli* as well as a deficient mutant in *C. glutamicum* have been observed to increase their oxygen consumption, suggesting that other bacteria are also not susceptible to back pressure^39^,^42^. Altogether, our data suggest that mycobacteria, like other genera of bacteria^43^,^44^, may have an energy spilling mechanism to modulate PMF and prevent excessive accumulation. Our measurements of membrane potential suggest that PMF modulation is sufficient to prevent the membrane from hyperpolarizing and causing back pressure, but unlike classical uncouplers does not go so far as to severely depolarize the membrane and thereby provide an additional impetus to increase metabolism and respiration. Without back pressure acting as a metabolic brake, BDQ's ATP depletion would be expected to result in the increase in respiration that we have observed (Fig. 1c), without the need to posit any other site or mechanism of action (Fig. 7b).

There are several possibilities for the precise mechanisms by which PMF is prevented from accumulating and causing back pressure with BDQ treatment. Less PMF might be generated by the ETC for each pair of electrons that traverse the ETC, either because of a switch to less efficient PMF-generating ETC complexes, or a because of the same complexes operating at a lower efficiency. Alternately, PMF might be dissipated by increased conductance of the membrane to protons or other charged species. These two mechanisms have previously been discussed respectively as intrinsic and extrinsic uncoupling^45^, or as decoupling and uncoupling^46^. Our experiments do not allow us to determine the relative contribution of intrinsic and extrinsic uncoupling to this phenomenon, which remains an area for further investigation. Further discussion of potential mechanisms of intrinsic and extrinsic uncoupling can be found in the Supplementary Discussion.

Mtb's regulation of its terminal oxidases has been discussed primarily in terms of differential expression^6^,^47^. Our data show that there is considerable rapid regulation of the enzymatic activity of existing proteins. Under real-time measurements, cytochrome bc_1_ inhibition causes rerouting of electron flux to cytochrome bd within a few minutes at most (Fig. 3b). It is unlikely that Mtb could react to this inhibition and produce new cytochrome complexes within this time frame. The promptness with which cytochrome bd is activated suggests that it must be activated through other means.

Regulation of cytochrome bd activity might be accomplished via allosteric regulation by the menaquinone pool. This is suggested by the fact that *C. glutamicum*'s closely related cytochrome bd is allosterically regulated by quinones at a second quinone binding site^47^. If cytochrome bd had more activity when its allosteric site is occupied by menaquinol as compared with menaquinone, the rapid switch from cytochromes bc_1_/aa_3_ to bd would be explained. This would allow preferential use of the energetically efficient cytochrome bc_1_/aa_3_ pathway when possible, but switch to cytochrome bd whenever bc_1_/aa_3_ failed to keep up with the supply of reducing equivalents and the menaquinone pool became more reduced. Mtb could then rapidly respond to changing O_2_ levels as well as respiratory inhibitors such as NO that are produced by host immune cells and to which cytochrome bd is less sensitive than aa_3_ (refs 48, 49). Given the rapidity with which cytochrome bd becomes active and the evidence of allosteric regulation in *C. glutamicum*, we propose that mycobacterial cytochrome bd activity level is allosterically regulated by the menaquinone pool.

BDQ and Q203 kill synergistically with CFZ by potentiating CFZ's ROS production. Of the types of biological stress we measured, ROS correlates best with the early rapid killing of these CFZ-containing combinations (Fig. 5d), and the antioxidant 4HT is protective (Fig. 5e). In our model BDQ and Q203 deplete ATP (Fig. 3d), increasing carbon catabolism and TCA cycle activity (Fig. 7d). As reducing equivalents accumulate faster than they can be oxidized by the ETC, they cause reductive stress (Fig. 5a). This potentiates CFZ's ability to transfer electrons from NDH2 to oxygen, increasing the generation of ROS and oxidative stress (Fig. 5c). Furthermore, it seems likely that this oxidative stress would be harder to tolerate by bacilli which are simultaneously starved of energy and thus limited in their ability to engage usual antioxidant defenses.

Research into ETC-targeting drugs is underway for other organisms, although to our knowledge, none have had as many ETC targets developed. For example, the *Staphylococcus* ETC has been shown to be targeted by clofazimine^50^, phenothiazines^51^ and diarylquinolones^52^. The elucidation of effective ETC combination-targeting strategies in Mtb may thus serve to inform strategies that can be used in other bacteria as more ETC-targeting agents are developed with different spectra of activity. Using the XF technology for studying Mtb has been particularly effective as it provides a single platform for studying the mode of action of ETC targeting drugs, genetic mutants of Mtb^27^, as well as toxicity against host cells.

For an obligate aerobe such as Mtb, the ETC fills an essential role in producing ATP, but also poses a substantial danger. We have shown here an example of combination-targeting that denies Mtb of the ATP generation benefits of the ETC, while also exploiting Mtb's metabolic flexibility to potentiate ETC-mediated ROS toxicity. These results present three valuable insights about energy metabolism targeting combinations not only in Mtb, but also potentially in other pathogens. First, the organism's response to disruption of energy metabolism may be complex due to extensive homoeostatic feedback loops. Second, targeting energy metabolism can work both by denying benefits of energy production and also by inducing damaging energy release. Third, effective combinations can exploit the response to one component of the combination to induce more damaging activity by another. Overall, this study provides crucial new evidence that the Mtb ETC can be an effective drug target, and it suggests that it may be most vulnerable to combination therapy. Finally, we anticipate a renewed interest in glycolysis as a potential drug target as disruption of both substrate level phosphorylation and OXPHOS will abolished ATP production, thereby leading to effective killing of persistent Mtb.

## Methods

### General

All Mtb strains were cultured in Middlebrock 7H9 media (Difco) supplemented with 10% OADC and 0.01% Tyloxapol at 37 °C, unless stated otherwise. Mtb H37Rv was obtained from BEI Resources (NR-123). Mtb mc^2^6230 (a non-pathogenic strain of Mtb auxotrophic for pantothenate and several amino acids) was a gift from Dr William Jacobs^53^. The Mtb *cydKO* mutant (a cytochrome bd knockout) was a gift from Dr Helena Boshoff^10^. BDQ was a gift from the Dr Digby Warner. The drug MIC_50_ values used during this study were as follows: BDQ 54 nM (ref. 54) (C_max_ 0.9–4.9 μM)^55^, Q203 3 nM (C_max_ 0.007–0.4 μM)^17^, CFZ 200 nM (C_max_ 1.1–4.1 μM although CFZ mostly accumulates in tissue at 1–3.6 mg g^−1^ of wet tissue in rats)^56^, rifampin 486 nM (ref. 57), ethambutol 2.45 μM (ref. 58), isoniazid 240 nM (ref. 59), streptomycin 172 nM (ref. 60) and moxifloxacin 1.14 μM (ref. 61). CellROX Orange, CellROX Green and DHE were purchased from Life Technologies; the ATP Bioluminescence Assay Kit CL II was obtained from Roche, protein concentration determination reagents from BioRad, and Cell-Tak from BD Biosciences. All other reagents were purchased from Sigma-Aldrich.

### Synthesis of Q203

Q203 ((6-chloro-2-ethyl-*N*-(4-(4-(4-(trifluoromethoxy)phenyl)piperidin-1-yl)benzyl) imidazo[1,2-*a*]pyridine-3-carboxamide) was prepared according to a reported literature method^17^,^62^. To a stirred solution of 4-(4-(4-(trifluoromethoxy)phenyl)piperidin-1-yl)phenyl)methanamine (1 mmol), 6-chloro-2-ethylimidazo[1,2-*a*] pyridine-3-carboxylic acid (1.1 mmol) in dry DMF and triethylamine (2 mmol) and 1-(3-Dimethylaminopropyl)-3-ethylcarbodiimide hydrochloride (1.5 mmol), 1-hydroxybenzotriazole (0.60 mmol) were added to the reaction mixture and stirring at 70 °C for 2 h. The reaction was quenched with water. The resulting solution was extracted with ethyl acetate and washed with sodium chloride. The organic layers dried over anhydrous magnesium sulfate and concentrated under vacuum and residue was loaded onto a silica gel column and eluted with Hexane/ethyl acetate (3:1) and then recrystallized from ethanol to obtain Q203 (60%) as a white solid, purity was ⩾95% and mass confirmed by LC-MS. ^1^H NMR (300 MHz, CDCl_3_): 9.52 (d, *J*=1.8 Hz, 1H), 7.51–7.54 (m, 1H), 7.30–7.24 (m, 5H), 7.15 (d, *J*=8.4 Hz, 2H), 6.98 (d, *J*=8.6 Hz, 2H), 6.04 (brt, *J*=5.4 Hz, 1H), 4.61 (d, *J*=5.4 Hz, 2H), 3.84–3.78 (m, 2H), 2.99 (q, *J*=7.7 Hz, 2H), 2.78–2.87 (m, 2H), 2.65–2.75 (m, 1H), 1.83–1.97 (m, 4H), 1.39 (t, *J*=7.5 Hz, 3H); LC-MS: *m/z* 557 [M+H]^+^.

### Mtb OCR and ECAR measurements

The OCR and ECAR of Mtb bacilli adhered to the bottom of a Cell-Tak coated XF cell culture microplate (Seahorse Biosciences), at 2 × 10^6^ bacilli per well, were measured using a XF96 Extracellular Flux Analyser (Seahorse Biosciences). Cell-Tak has no effect on Mtb basal respiration (Supplementary Fig. 9). Assays were carried out in unbuffered 7H9 media (pH 7.35) without carbon source. Mtb bacilli were starved in liquid media, using 7H9 supplemented only with 0.01% Tyloxapol, for 24 h before being seeded into the XF cell culture microplate and the start of the experiment. In general, basal OCR and ECAR were measured for ∼21 min before the automatic addition of the either glucose (single drug and drug combination experiments) or the ETC-targeting drugs (long exposure experiment) through the drug ports of the sensor cartridge. During the long exposure experiment, bacilli were seeded into the microplate wells using media containing 2 mg ml^−1^ glucose and it was thus not added during the assay. In the single drug and drug combination experiments the ETC-targeting drugs (single or combinations) were added ∼21 min after glucose. The length of OCR and ECAR measurement after ETC-targeting drugs addition and the concentration used depended on the experiment done. The assays concluded with the further addition of 2 μM CCCP (final concentration) after which OCR and ECAR were measured for an additional 21–35 min. All OCR and ECAR figures indicate the point of each addition (glucose, drug, CCCP) as dotted lines. OCR and ECAR data points are representative of the average OCR and ECAR during 4 min of continuous measurement in the transient microchamber, with the error being calculated from the OCR and ECAR measurements taken from three replicate wells by the Wave Desktop 2.2 software (Seahorse Biosciences). The transient microchamber is automatically re-equilibrated between measurements through the up and down mixing of the probes of the XF96 sensor cartridge in the wells of the XF cell culture microplate.

To measure Mtb OCR as a function of pO_2_ the probes of the XF96 sensor cartridge were allowed to settle down and maintain the transient microchamber for 4 h at a time after ETC-targeting drug and media control addition. This allowed for OCR measurements in the absence of re-equilibration of the transient microchamber. OCR was calculated for a moving window of 5 individual measurements representing ∼1 min, and curves were constructed describing OCR as a function of pO_2_.

### ROS assay

Mtb mc^2^6230 was cultured using 7H9 media supplemented OADC, 0.01% Tyloxapol, 2 mg ml^−1^ casamino acids and 24 μg ml^−1^ pantothenate. ROS production in Mtb mc^2^6230 after drug exposure was measured using the following three ROS sensing dyes: DHE (Ex/Em of 518/605 nm), CellROX Orange (Ex/Em of 545/565 nm) and CellROX Green (Ex/Em of 485/520 nm). Cultures were filtered through a 10 μm filter to generate a single cell suspension and drug treated at 300 times the MIC_50_ concentration, for 1 h. Cumene hydroperoxide at 15 mM and CCCP at 2 μM were used as positive and negative controls respectively. The ROS sensing dyes were added to the treated bacilli (DHE at 10 μM, CellROX Orange and Green at 5 μm) and incubated for a further 30 min. Before analysis, the treated bacilli were pelleted and resuspended in fresh media to remove any residual extracellular dye. Samples were acquired using a Guava EasyCyte 8HT flow cytometer (Millipore) using the ExpressPlus Module, collecting 5,000 events at a flow rate of 0.59 μl s^−1^. Median fluorescent intensity was calculated with the FlowJo software package.

The fluorescence of the stained sample was acquired and analysed with the FACSAria III cell sorter (Special Ordered Research Product). The fluorescence of DHE were acquired using the 488 nm laser excitation, and BP 610/20 nm for emission acquisition. The cells were acquired at a constant flow rate of setting 4 and the threshold rate of approximately 2,000–3,000 events per second and 1,000,000 the total events were recorded per sample. For result analysis, the bacterial population was identified according to the forward and side light scattering property of the population (FSC versus SSC). The doublet bacteria are discriminated from the analysis according to the relationship of the height and area of the FSC signal pulses of the bacteria. Median fluorescent intensity was calculated with the FlowJo software package.

### IMV preparation

Mtb mc^2^6230 was grown aerobically at 37 °C in 7H9 media enriched with 10% OADC, 0.2% casamino acids, 24 μg ml^−1^pantothenate, and 0.01% tyloxopol. Growth was monitored via absorbance at 600 nm. When the OD reached ∼0.8, bacteria were harvested and IVMs made by essentially the method described by Yano *et al*^19^. The technique was modified as follows. Bacteria were lysed by bead beating in a Roche MagNA Lyser at 7,000 r.p.m. for 1 min repeated for six cycles, with 5 min of cooling on ice between cycles. The final centrifugation step was performed in an F0630 rotor in an Allegra 64R centrifuge at 59,860 × g for 210 min. After snap-freezing in liquid nitrogen, aliquots were stored at −80 °C until use. Protein content was estimated at 14 mg ml^−1^by reaction with Bradford Reagent. Dithionite reduced minus oxidized spectrum had peaks corresponding to cytochromes *b*, *c* and *a*, consistent with aerobic growth conditions.

### IVM experiments

All IMV experiments were carried out in the same buffer that they had been prepared in (10 mM HEPES, 50 mM KCl, 5 mM MgCl_2_, 10% glycerol, pH 7) unless otherwise specified at 37 °C. All figures show averages and standard deviations of triplicate repeats. Statistical significance was calculated with GraphPad Prism 6.

ATP synthesis was measured using luciferase/luciferin from Roche Bioluminescence Assay Kit CL II. Luciferase/luciferin mix was combined with equal portions of buffer, with IMVs yielding a final concentration of 35 μg ml^−1^ membrane proteins, 250 μM NADH, 50 μM ADP and 5 mM phosphate. Luminescence was monitored on a BioTek Synergy H4 plate reader in a 384-well plate. Positive control contained no other additions, IMVs in drug treatment wells were pre-incubated with drugs for ∼10 min, and the negative control lacked NADH. The rate of ATP synthesis was calculated from the rate of change of luminescence over the period from 15–30 min after combination of reagents.

NADH consumption was measured in an Agilent Cary 100 UV-Vis spectrophotometer. 250 μM NADH, 50 μM ADP and 5 mM P_i_, were added to a quartz cuvette, and absorbance was monitored at 340 nm. The rate of NADH consumption was measured between sequential additions of IMVs to a final concentration of 0.05 mg ml^−1^, drug or DMSO control, and finally 20 mM KCN. NADH consumption was calculated as a fraction of activity of that of the DMSO-treated control.

ΔpH generation was measured using the fluorescent dye 9-amino-6-chloro-2-methoxyacridine (ACMA). IMVs totalling 140 μg ml^−1^ of protein were combined with 500 μM NADH, 50 μM ADP, 5 mM phosphate and 4 μM ACMA. Treatment group IMVs were pre-incubated with drugs for ∼10 min before addition of NADH. The positive control had no drug treatment; the negative control lacked NADH. Fluorescence was measured on a BioTek Synergy H4 plate reader with excitation at 412 nm and emission at 510 nm.

Proton conductance was also measured with ACMA, using a similar method to that described by Pringle^63^. IMVs totalling 140 μg ml^−1^ of protein were added to buffer containing NaCl instead of KCl, with 4 μM ACMA and the indicated concentrations of tested drugs. A ΔΨ was established by addition of 20 μM valinomycin; ACMA fluorescence quenching 30 s later was taken to represent proton conductance.

### Measuring the change in membrane potential

The change in membrane potential of drug-treated H37Rv bacilli was determined using the BacLight Bacterial Membrane Potential Kit (ThermoFicher Scientific) according to the manufacturer's instruction. In short, five separate H37Rv cultures, at an OD_600_ of ∼0.8, were filtered through a 10.0 μm syringe filter (Pall Life Sciences) to obtain a single cell suspension. Culture aliquots (1 ml) were treated with 20 μM CCCP and 300 × MIC_50_ concentrations of BDQ, Q203 and CFZ, respectively, after the addition of the fluorescent dye DiCO_2_ (3 μM). DMSO-treated and -untreated bacilli were used as controls. Aliquots were incubated for 30 min before analysis on a FACSAria III.

### Kill kinetics of the drug combinations on Mtb

Mid-log phase Mtb H37Rv was diluted to an OD_600_ of 0.01 in 7H9 media (2 mg ml^−1^ glucose, 0.01% Tyloxapol). Individual cultures were treated with the single and combination ETC-targeting drugs at 30 times their respective MIC_50_ concentrations. Samples were taken at indicated time points, serially diluted in phosphate buffered saline, and plated onto 7H11 OADC agar plates. Combinations were considered synergistic if they exhibited a >=2 log_10_(CFU per ml)-fold decrease as compared with the most active single agent at any time point^64^.

For kill kinetics in the presence of antioxidants, mid-log phase Mtb H37Rv was diluted to an OD_600_ of 0.01 in catalase free 7H9 media (2 mg ml^−1^ glucose, 0.01% Tyloxapol). Triplicate cultures were independently treated with the BDQ/Q203/CFZ combination at 30 × MIC_50_ concentrations, in the presence of 5 mM concentrations of the antioxidants 4-Hydroxy-2,2,6,6-tetramethylpiperidyl-1-oxy (4-OH-TEMPO) and NAC, respectively^19^. Samples were taken 5 days after drug addition, serially diluted in phosphate buffered saline, and plated onto 7H11 OADC agar plates.

### ATP/ADP/AMP and NADH/NAD^+^ ratios in Mtb lysates

Mtb H37Rv cultures were treated with 30 times the MIC of BDQ, Q203, CFZ, BDQ/Q203, BDQ/CFZ, Q203/CFZ, BDQ/Q203/CFZ and RIF/INH. At the specific time points 1.8 ml of each culture was pelleted in triplicate, 1 ml of cold chloroform:methanol at a ratio of 2:1, (containing the internal standard, 6-amininicotinic acid) was added and stored at −80 °C until further processing. An additional 1 ml of each culture was pelleted and formalin fixed for weight determination. During further processing the bacilli lysate chloroform:methanol mixture was spun through a 0.22 μM filter and the filtrate evaporated under reduced pressure, while centrifuged. The dried pellet was reconstituted in 200 μl water overnight and submitted for LC-MS analysis. The ATP/ADP/AMP and NADH/NAD^+^ ratios were determined with two different LC-MS/MS methods. For the NADH/NAD^+^ ratios the analytes were separated on a Zorbax Eclipse Plus C18 column (2.1 × 100 mm, 3.5 μm) using a gradient elution programme at a flow rate of 200 μl min^−1^ and an injection volume of 10 μl. Two mobile phases were used: mobile phase A (A, 0.1% formic acid in water) and mobile phase B (B, 0.1% formic acid in acetonitrile). The gradient programme was as follows: 0–5 min, a linear increase from 0–90% of B; 5–15 min, 90% of B; 15–15.1 min, 0% of B; and 15.1–27 min, 0% of B. For NADH the precursor ion monitored in positive mode was 666 *m/z* with the two product ions being 649 *m/z* and 107.8 *m/z*, for NAD^+^ 664 *m/z* was monitored with the two product ions 427.9 *m/z* and 135.9 *m/z*, respectively. For the ATP/ADP/AMP ratios the analytes were separated on a Waters Exterra C18 column (2.1 × 100 mm, 3.5 μm) using a gradient elution programme at a flow rate of 200 μl/min and an injection volume of 5 μl. Two mobile phases were used: mobile phase A (A; 10 mM tributylamine, 15 mM acetic acid in 3% acetonitrile, 97% water) and mobile phase B (B; 10 mM tributylamine, 15 mM acetic acid in 100% acetonitrile). The gradient programme was as follows: 0–1 min, 0% of A; 1–10 min, a linear increase from 0–100% of B; 10–15 min, 100% of B; 15–15.1 min, 0% of B; and 15.1–25 min, 0% of B. The ATP/ADP/AMP precursor ion/product ions monitored were the following, respectively: 505.9 *m/z*, and 78.8 *m/z* and 159 *m/z*; 425.9, and 78.9 *m/z* and 133.9 *m/z*; 345.9 *m/z*, and 78.9 *m/z* and 96.8 *m/z*. The AUC were normalized to dry weight bacilli before ratios were determined.

### RAW264.7 and HepG2 cell bioenergetic measurements

RAW264.7 (ATCC TIB-71) and HepG2 (ATCC HB-8065) cells cultured in modified DMEM media (containing 4.5 g l^−1^ glucose, 3.7 g l^−1^ sodium bicarbonate, 870 mg l^−1^ glutamine, 110 mg l^−1^ sodium pyruvate and supplemented with 10% FBS) were seeded into a XF96 cell culture microplate at densities of 65,000 and 25,000 cell per well, respectively, and incubated overnight in a CO_2_ incubator at 37 °C. The supernatant was removed the following day and replaced with 180 μl Seahorse Assay media (unbuffered, containing 4 g l^−1^ glucose) and incubated at 37 °C in a CO_2_-free incubator for 1 h before the start of the respective assay. Three basal measurements were taken before addition of the different drug combinations (at 30 × their respective MIC_50_ values), after which basal OCR was measured for 1 h. During the ‘modified Cell Mito Stress assay' three basal measurements were taken before the addition of the Mtb ETC targeting drugs, followed by an additional 3 measurements. First, oligomycin was added (3.0 μM and 1.5 μM to HepG2 and RAW264.7 cells, respectively) to assess the effects of the Mtb ETC drugs on ATP turnover in these cell lines. Next, FCCP (final concentration of 1.5 μM) was added to determine the effect spare respiratory capacity. Finally, rotenone and antimycin A, both at a final concentration of 0.5 μM, were added to determine drug effects on non-mitochondrial respiration.

### RAW264.7 and HepG2 cell viability assay

RAW264.7 and HepG2 cells cultured in modified DMEM were seeded into a 96 well culture plated and incubated overnight at a CO_2_ incubator at 37 °C. The following day, after removal of the supernatant fluid, modified DMEM media (without phenol red) containing the different drug combinations was added, and the cells were incubated for a further 2 days, after which the MTT (Vybrant MTT Cell Proliferation Assay Kit, Thermo Scientific) assay was performed according to the manufacturer's instructions.

### Effect of drug combinations on Mtb in macrophages

RAW264.7 macrophages cultured in modified DMEM media were seeded into 12-well cell culture plates, at a cell density of 1 × 10^5^ cells per well, and incubated overnight in a CO_2_ incubator at 37 °C. RAW264.7 macrophages were infected with mid-log Mtb H37Rv at MOI (multiplicity of infection) of 1. After 4 h of infection the macrophages were washed with media to remove the extracellular Mtb, after which media containing the different drug combinations was added. At the specific time points, the macrophages were lysed with a final concentration of 0.1% SDS and the lysates were serially diluted in PBS containing 0.02% Tween-80 and plated onto 7H11 OADC agar plates containing PACT for CFUs. Three independent experiments were performed in triplicate.

### Data availability

The authors declare that the data supporting the findings of this study are available within the article and its Supplementary Information Files, or from the corresponding author on request.

## Additional information

**How to cite this article:** Lamprecht, D.A. *et al*. Turning the respiratory flexibility of *Mycobacterium tuberculosis* against itself. *Nat. Commun.* 7:12393 doi: 10.1038/ncomms12393 (2016).

## Supplementary Material

Supplementary Information Supplementary Figures 1-9, Supplementary Discussion and Supplementary References
