# Supplementary material for: Turning the respiratory flexibility of Mycobacterium tuberculosis against itself
Source: Nat Commun. 2016 Aug 10;7:12393. doi: 10.1038/ncomms12393 (PMC4987515; doi:10.1038/ncomms12393)
Supplement: Supplementary Information — Supplementary Figures 1-9, Supplementary Discussion and Supplementary References [file ncomms12393-s1.pdf]

Supplementary Information

Supplementary Figures

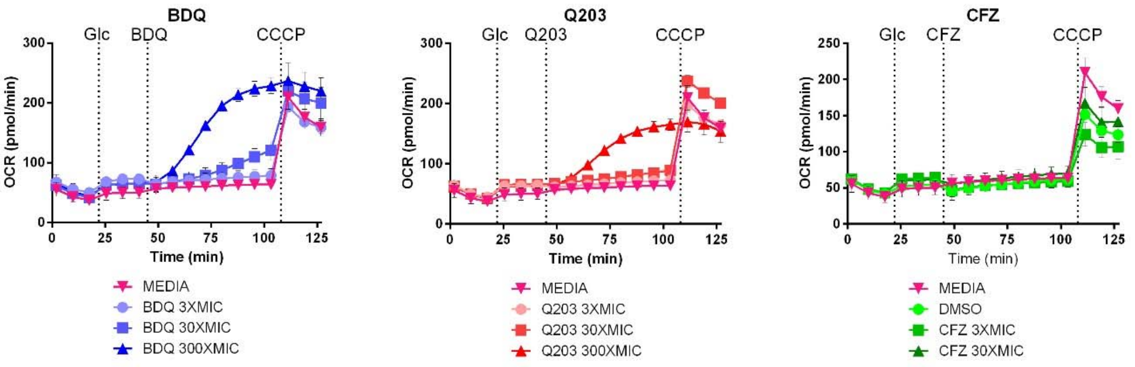

Supplementary Figure 1: The absolute OCR profiles for the data presented in Figure 1. The vertical dotted lines indicate the time points at which the glucose (Glc), the specific drug (at the respective concentrations) and CCCP was injected. *Mtb* H37Rv treated with BDQ (3x, 30x and 300x the MIC_{50}), Q203 (3x, 30x and 300x the MIC_{50}) and CFZ (only at 3x and 30x the MIC_{50} due to lack of solubility at 300x) in the presence of glucose as carbon source. DMSO was used as a vehicle control. One representative experiment is shown, three replicate experiments were performed.

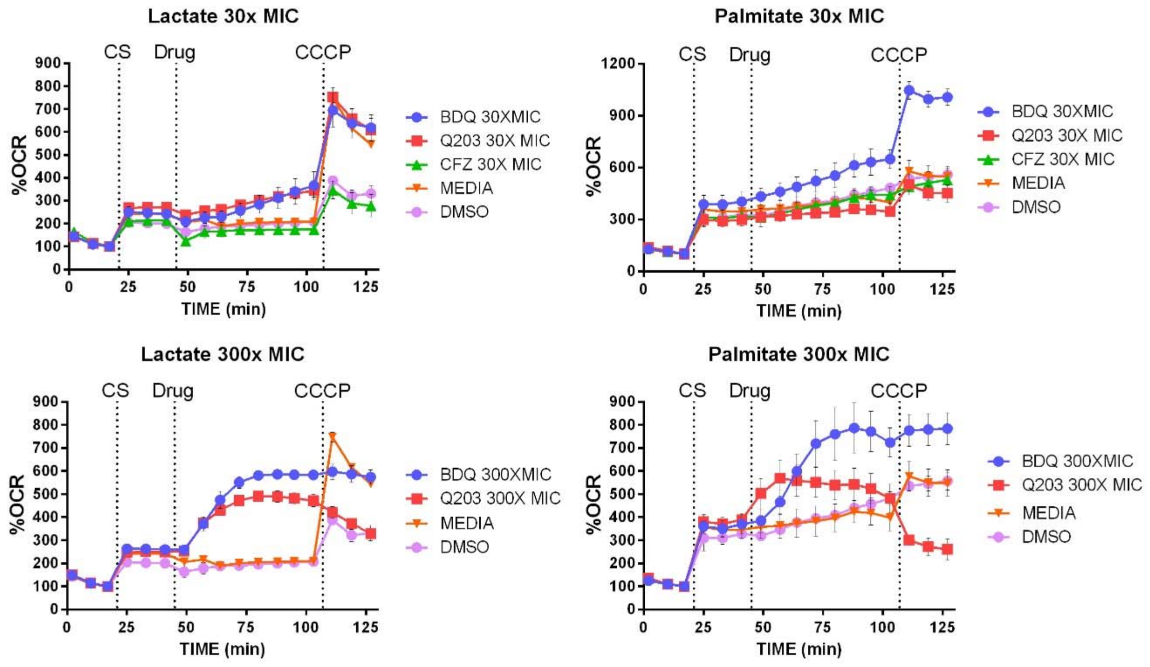

Supplementary Figure 2: The OCR profiles of *Mtb* H37Rv treated with BDQ (x30 and x300 the MIC_{50}), Q203 (x30 and x300 the MIC_{50}) and CFZ (x30 the MIC_{50}) in the presence of lactate and palmitate as carbon sources. The vertical dotted lines indicate the time points at which the carbon source (CS), the specific drug and CCCP was injected. It is clear from the OCR profiles that the increase in OCR after BDQ and Q203 addition is not carbon source dependent. One representative experiment is shown, three replicate experiments were performed.

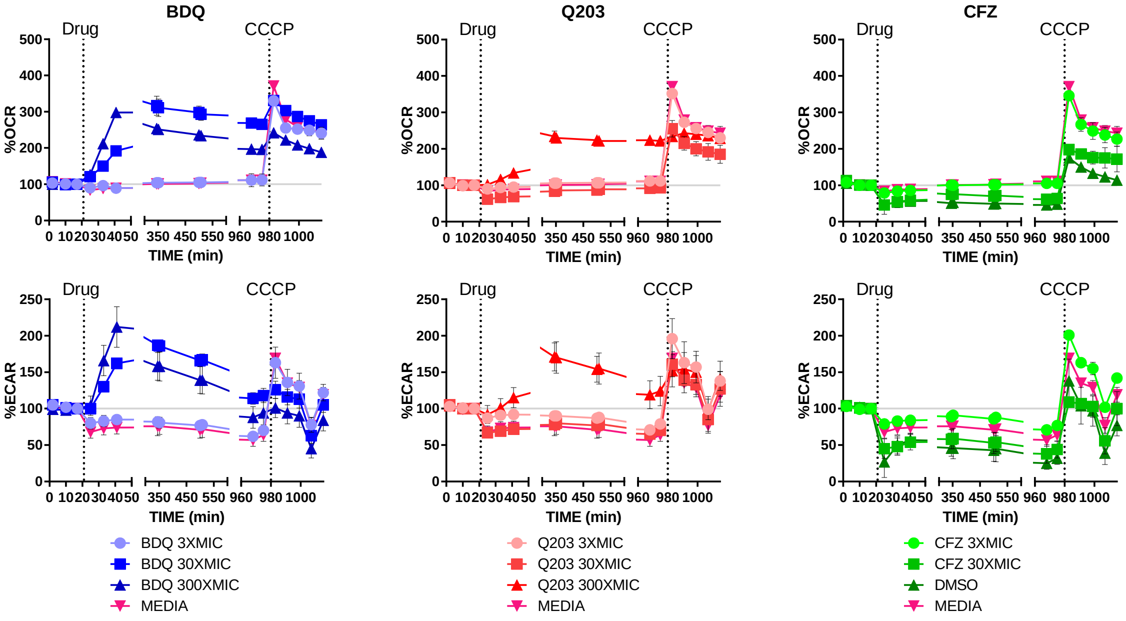

Supplementary Figure 3: The extended (~16 hrs) OCR and ECAR profiles of *Mtb* H37Rv treated with BDQ, Q203 and CFZ in the presence of glucose as a carbon source. The vertical dotted lines indicate the time points at which the specific drug and CCCP was injected. The profiles indicate that increase in both OCR and ECAR, after BDQ and Q203 addition, is maintained for at least 16 hrs after addition, and after the addition of CCCP the bacilli were still able to increase their OCR and ECAR. One representative experiment is shown, two replicate experiments were performed.

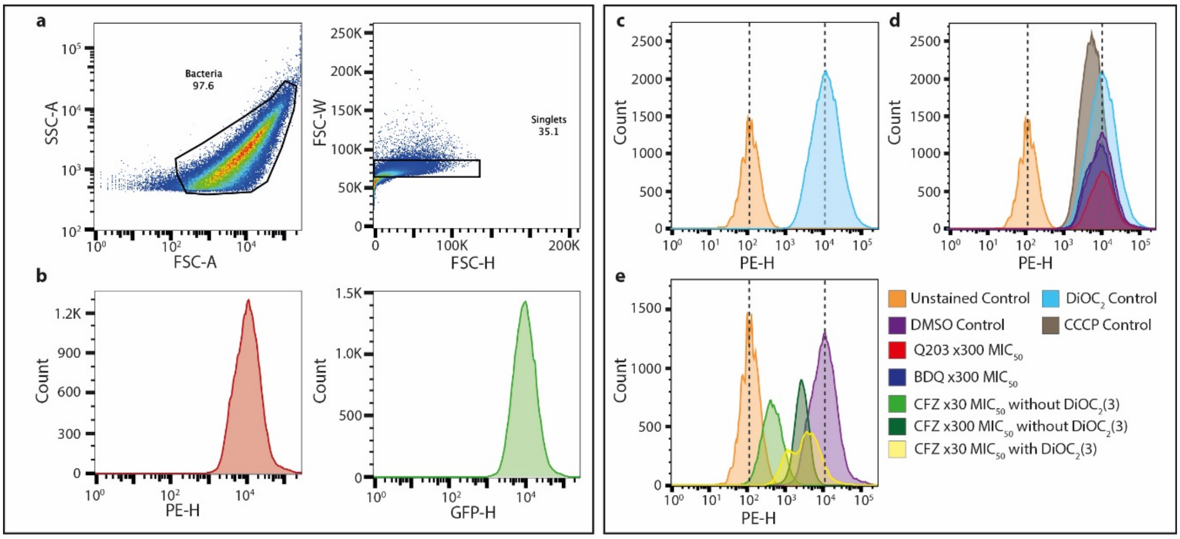

Supplementary Figure 4: Gating scheme and workflow used to determine the effect of BDQ, Q203 and CFZ on *Mtb* H37Rv membrane potential. **(a)** Gating scheme used to analyze red/green fluorescence of individual *Mtb* bacilli. **(b)** MFI of red (PE) and green (GFP) fluorescence of gated *Mtb* cells used to calculate the red:green ratio. DIOC$_2$(3) self-associates in healthy cells to emit red fluorescence; when membrane potential decreases, there is a shift in red fluorescence to green. **(c)** MFI of (PE) in unstained *Mtb* compared to DIOC$_2$(3)-stained control cells. **(d)** The shift in DIOC$_2$(3) fluorescence after CCCP addition relative to BDQ, Q203 and DMSO control after 30 min. **(e)** CFZ is a red iminophenazine that could not be separated from DIOC$_2$(3) in the PE channel. MFI, mean fluorescence intensity; PE, phycoerythrin; GFP, green fluorescent protein; BDQ, bedaquiline; CFZ, clofazimine.

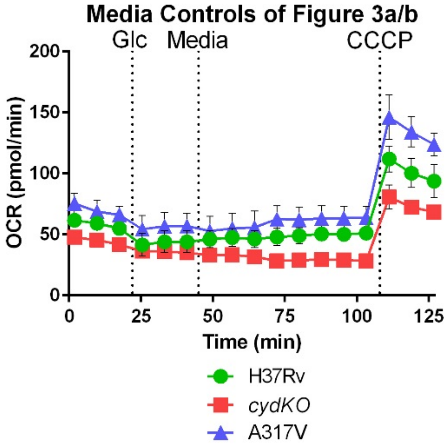

Supplementary Figure 5: The OCR profiles of the untreated media controls of the H37Rv, *cydKO* and A317V strains used in Figure 3a and b. The vertical dotted lines indicate the time points at which the glucose (Glc), no drug containing nor carbon source containing media and CCCP (final concertation of 2 µM) were injected. All three strains show the same relative increase in respiration after CCCP addition. One representative experiment is shown, two replicate experiments were performed.

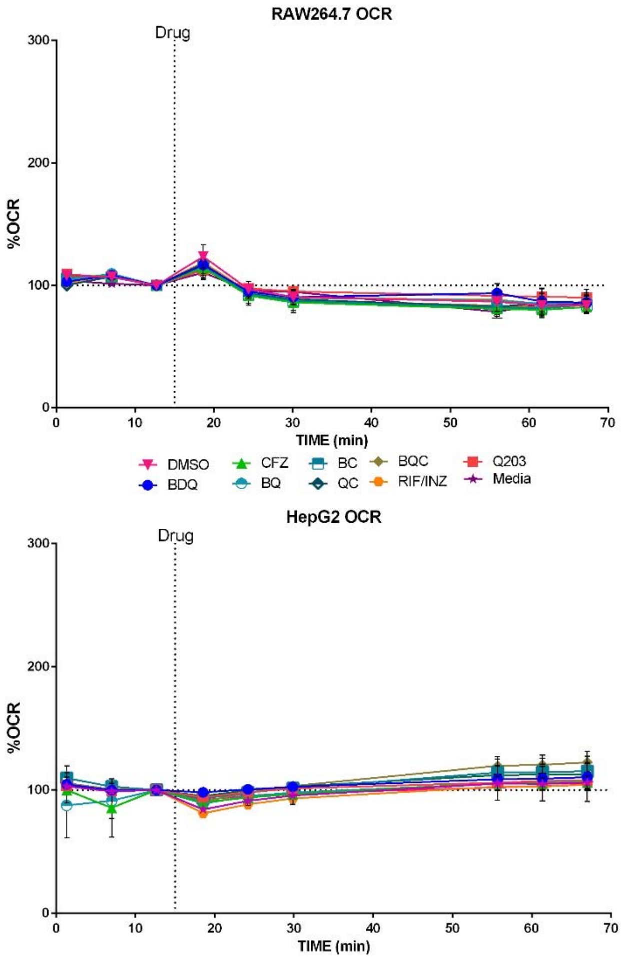

Supplementary Figure 6: Basal respiration bioenergetic profiles of RAW264.7 and HepG2 cell lines after the addition of the different *Mtb* ETC targeting drug combinations. RAW264.7 cells (top panel) and HepG2 cells (bottom panel) were seeded at a density of 65,000 and 25,000 cells per well, respectively, and, basal respiration was measured prior to treatment with the *Mtb* ETC targeting drug combinations at 30x their MIC_{50} concentrations as indicated by the vertical dotted line. Basal respiration was measured for 1 hr after drug addition, during which time there was no significant differences between the ETC targeting drug treated cells and the various controls (media, DMSO, RIF & INH).

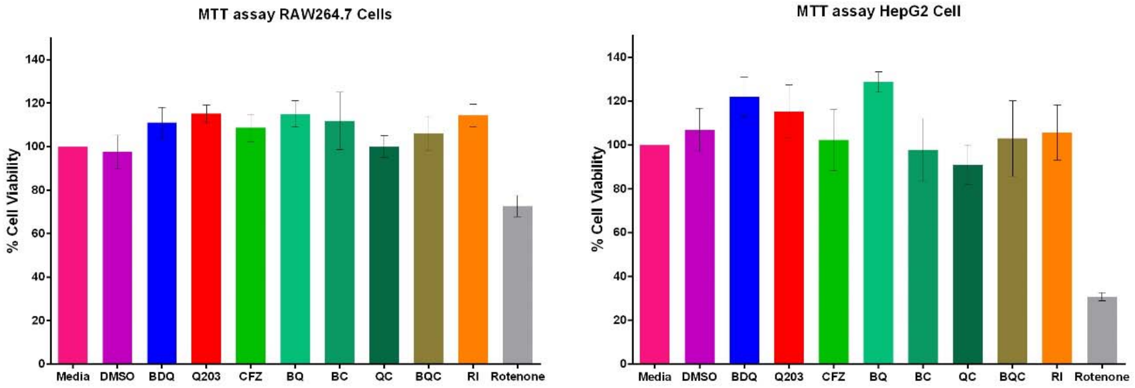

Supplementary Figure 7: MTT cell viability assay. RAW264.7 and HepG2 cells were seeded into a 96 well plate at a density of 2 x 10^4 cells per well and treated with the different drug combinations at 30x their MIC_{50} concentrations for 48 hours. Rotenone was used at 2.5 µM for the RAW264.7 control and 125 µM for the HepG2 cell control. The MTT (Vybrant® MTT Cell Proliferation Assay Kit, Thermo Scientific^{TM}) assay was preformed according to the manufacturer's instructions. Data shown are representative of three independent experiments. Mean and standard errors were calculated from four individual wells of each treatment.

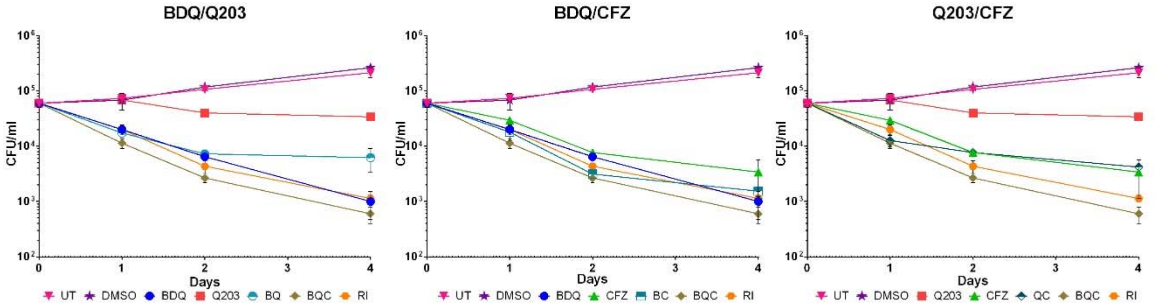

Supplementary Figure 8: Killing efficiency of different *Mtb* ETC- targeting drug combinations using a macrophage model. Kill curves were constructed by infecting 200,000 RAW264.7 cells per well with *Mtb* H37Rv at a MOI of 1. Cells were drug-treated at 30X MIC_{50} with the indicated drugs for four days. Data shown is a representative set of three independent experiments. Mean and standard errors were calculated from three individual cultures for each treatment condition.

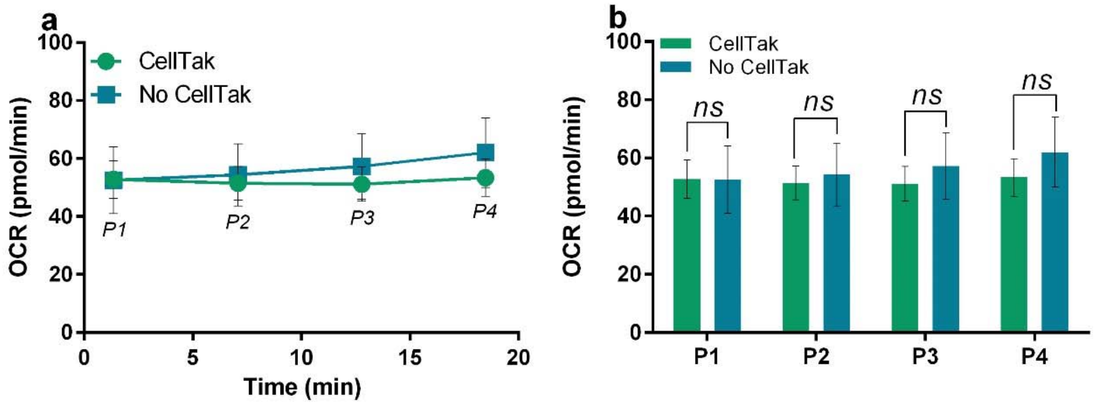

Supplementary Figure 9: The effect of Cell-Tak on *Mtb* H37Rv basal respiration. *Mtb* H37Rv bacilli were seeded into both Cell-Tak coated and non-coated wells of the XF cell culture microplate at a density of 2 x 10^6 bacilli per well and centrifuged as described in the Materials and Methods section. **(a)** The OCR profiles of H37Rv bacilli measured with and without the use of Cell-Tak. Basal respiration was measured for four readings over ~20 minutes. **(b)** There is no statistical difference between the OCR measurements taken in the presence/absence of Cell-Tak at each of the four basal respiration readings (*P1-P4*), although the non-Cell-Tak bacilli OCR measurements standard deviation is on average 50% higher than that of the Cell-Tak bacilli OCR measurements. This demonstrates the advantage of using Cell-Tak to adhere the bacilli to the XF cell culture microplate. The experiment was done in duplicate with the mean and standard deviations calculated from nine replicate wells in each experiment (n=9). Unpaired t-test (using GraphPad Prism 6.05) was used to analyze the statistical difference between each of the four basal respiration readings.

Supplementary Discussion

Mtb ETC targeting drug combination cytotoxicity

Previous studies indicate that BDQ, Q203 or CFZ as monotherapy did not have any cytotoxic effects on mammalian cells when they are treated with the same concentrations used in our study[1-3], but the combined effect of these *Mtb* ETC targeting drugs on mammalian respiration has not yet been determined. XF technology has been frequently used to assess drug toxicity and is being regarded as superior to purified mitochondria as it provides a cellular context[4-6]. Here, in addition to the conventional approach that measures the effects of drugs on unstressed mitochondria, we determine if the drug combinations had an effect on mammalian bioenergetics by examining four different bioenergetic parameters in HepG2 (Figure 6, panels a-c) and RAW 264.7 (Figure 6, panels d-e) cell lines. Firstly, the drug combinations had no effect on basal respiration (BR) above that of the media, DMSO and RIF/INH controls; indicating that when these drugs are used in combination they do not affect normal cellular respiration. BR of drug treated cells does not deviate from the controls' basal respiration even after extended drug treatment (Supplementary Fig. 6).

Secondly, ATP turnover (AT) is the decrease observed in OCR upon addition of oligomycin, which blocks the proton channel of the F_0-subunit of mammalian ATP synthase and inhibits ATP synthesis. Although the CFZ drug combinations induced a slightly smaller AT in the HepG2 cell line than the non-CFZ drug combinations, the difference was minute. One possible explanation for the smaller HepG2 AT in the presence of CFZ (a FDA approved drug) might be that the CFZ concentrations examined induce a slight inhibitory effect on HepG2 NADH dehydrogenase, which would decrease the electron flux into the HepG2 ETC and lower the AT. This effect was not seen in the RAW 264.7 where all drug combinations induced a similar AT.

Thirdly, mammalian cell spare respiratory capacity (SRC) was calculated from the difference between the BR and the maximal respiration induced following the addition of the uncoupler carbonyl cyanide-p-trifluoromethoxyphenylhydrazone (FCCP). Although there are small differences in the SRC between the different drug combinations in the HepG2 cell line, they are all centered between the SRC of the media and DMSO controls, indicating these minor differences are due to the DMSO used to dissolve the drug combinations and not the drug combinations themselves. In the RAW 264.7 cell line there were no major differences between the drug combinations and controls.

Lastly, non-mitochondrial respiration (non-MR) was measured after the addition of rotenone and antimycin A to completely shut down mitochondrial OCR by inhibition of Complexes I and III, respectively. No differences were observed between the drug combinations and controls, in both the HepG2 and RAW 264.7 cell lines. This demonstrates that the drug combinations are not targeting any

other cellular oxygen consuming processes, for example NADPH oxidase, which is important for pathogen killing via ROS production[7].

Finally, to independently determine if the *Mtb* ETC targeting drug combinations have an effect on mammalian cell viability we performed a MTT (3-(4,5-dimethylthiazol-2-yl)-2,5-diphenyltetrazolium bromide) assay on both RAW 264.7 and HepG2 cells, which were treated with the drug combinations for two days (Supplementary Fig. 7). No differences were found between the drug combinations and the media, DMSO and RIF/INH controls. However, there was a 23 % and 96 % reduction in the viability of RAW 264.7 and HepG2 cells, respectively, when treated with the control rotenone over the same time period. Thus, the *Mtb* ETC targeting drug combinations have no effect on host cell viability.

In sum, the *Mtb* ETC targeting drug combinations did not alter the BR, AT, SRC, non-MR or viability of the mammalian cell lines HepG2 and RAW 264.7, indicating that the combinations do not target the mammalian ETC under unstressed or stressed conditions, or have any off-target effects leading to obvious cytotoxicity.

Potential mechanisms of intrinsic or extrinsic uncoupling

If intrinsic uncoupling is responsible for lack of observed back pressure with BDQ treatment, it would likely consist of PMF-generating complexes slipping and failing to translate protons across the membrane even as electron flux continues, because our experiments (Figure 3a) suggest that BDQ treatment does not reroute electrons through less efficient PMF-generating ETC complexes. In other systems, proton-pumping complexes slip in a potential-dependent fashion and have been proposed to result in a fixed maximum achievable PMF[8]. This would be consistent with our observations, as even when consumption of PMF by ATP synthase is fully inhibited, the *Mtb* ETC does not drive PMF above a fixed value.

Extrinsic uncoupling could take several forms. One might be for BDQ to directly cause an increase in proton conductance, by binding to the subunit a-c interface of ATP synthase subunit F_0, allowing it to act as a proton channel, as has recently been proposed by other authors[9]. We argue that this is unlikely for several reasons. First, our direct measurements of proton conductance in IMVs show no change with BDQ (Figure 2c). Second, BDQ does not reduce membrane potential in live cells, unlike the protonophore CCCP (Figure 2h). Third, DCCD increases OCR similarly (Figure 3c), suggesting that increased OCR is a common downstream response to varied types of Complex V inhibition. Fourth, the recently completed crystal structure of the F_0 c-ring with BDQ shows BDQ binds on the H^+ binding site and prevents the c-ring from turning[10]. It is unlikely that BDQ has two binding sites on ATP

synthase with two distinct effects. Together, these findings suggest that the increase in OCR cannot be attributed to this proposed mechanism.

If extrinsic uncoupling is responsible for the lack of observed back-pressure in BDQ treated *Mtb*, it is more likely a downstream consequence of inhibiting ATP synthase rather than a direct effect of the drug. Classical uncouplers, such as CCCP and mitochondrial Uncoupling Protein, decrease membrane potential in live cells and increase membrane conductivity in membrane vesicles[11]. However, neither effect was observed when we treated *Mtb* with BDQ (Figures 2c and 2h), suggesting a different dynamic. Other authors have posited that mitochondria may have a "mild uncoupling" mechanism: when ATP synthase activity is low due to low ATP turnover, then non-ohmic conductance dissipates any PMF in excess of a maximum safe value[12]. This would be more consistent with our experimental data.

Supplementary References

1 Clofazimine. *Tuberculosis* **88**, 96-99, (2008).

2 TMC-207. *Tuberculosis* **88**, 168-169, (2008).

3 Kang, S. *et al.* Lead optimization of a novel series of imidazo[1,2-α]pyridine amides leading to a clinical candidate (Q203) as a multi- and extensively-drug-resistant anti-tuberculosis agent. *J. Med. Chem.* **57**, 5293-5305, (2014).

4 Beeson, C. C., Beeson, G. C. & Schnellmann, R. G. A high throughput respirometric assay for mitochondrial biogenesis and toxicity. *Anal. Biochem* **404**, 75-81, (2010).

5 Rana, P., Anson, B., Engle, S. & Will, Y. Characterization of human-induced pluripotent stem cell–derived cardiomyocytes: bioenergetics and utilization in safety screening. *Toxicol. Sci* **130**, 117-131, (2012).

6 Wills, L. P. *et al.* High-throughput respirometric assay identifies predictive toxicophore of mitochondrial injury. *Toxicol. Appl. Pharmacol.* **272**, 490-502 (2013).

7 Segal, A. W. The function of the NADPH oxidase of phagocytes and its relationship to other NOXs in plants, invertebrates, and mammals. *Int. J. Biochem. Cell Biol.* **40**, 604-618, (2008).

8 Nelson, N., Sacher, A. & Nelson, H. The significance of molecular slips in transport systems. *Nat. Rev. Mol. Cell Biol.* **3**, 876-881, (2002).

9 Hards, K. *et al.* Bactericidal mode of action of bedaquiline. *J Antimicrob Chemother*, (2015).

10 Preiss, L. *et al.* Structure of the mycobacterial ATP synthase F_o rotor ring in complex with the anti-TB drug bedaquiline. *Sci. Adv.* **1**, e1500106, (2015).

11 Klingenberg, M., Winkler, E. & Echtay, K. Uncoupling protein, H^+ transport and regulation. *Biochem. Soc. T.* **29**, 806-811, (2001).

12 Skulachev, V. P. Uncoupling: new approaches to an old problem of bioenergetics. *Biochim. Biophys. Acta* **1363**, 100-124, (1998).
